# Surgery

**Published:** 1893-06

**Authors:** W. H. Harsant


### SURGERY.

Operations for the radical cure of hernia have now become so frequent that in some of the large hospitals it is said that an operating day seldom passes without one or more of these being on the programme. Nothing very new has been introduced in the principles of operating since Bassini described[2] his method of lifting the spermatic cord out of the inguinal canal, and making a new track for it at a higher level; but various improvements have been suggested in the details of operating, chiefly in the direction of making the operation more thorough and

[2] *Nuovo metodo operativo per la cura dell' ernia inguinale*, 1889. See also *An. Univ. Med. Sci*,, 1892, vol. iii., C—109.

radical, while there is also a general consensus of opinion that drainage should be as far as possible dispensed with. Among recent papers, the following are perhaps the most important:

Dr. G. R. Fowler, of Brooklyn, in a paper read before the New York Surgical Society,[1] points out that the most conservative of advanced surgeons now agree that a hernia which cannot be retained easily, painlessly, and with certainty, by means of a truss, should be operated upon, with a view of bringing about a radical cure. Those less conservative assert that a hernia which increases in size rapidly, thus requiring frequent changes of size and shape, as well as amount of pressure exercised by a truss, should be subjected to the operation for radical cure. Also that the operation of herniotomy should always include an attempt at radical cure. It would certainly be an anomaly at the present day to witness an operation for the relief of strangulated hernia, in which the surgeon failed to make the attempt, at least, to give the patient the benefit of whatever means were at hand to prevent the recurrence of the accident.

The slight additional time occupied and traumatism inflicted can have no weight when placed in the balance with the incalculable benefit to be derived from the radical cure. Under these circumstances the prognosis of the operation for radical cure becomes the prognosis of the herniotomy for necessity, and nothing more.

The prognosis as regards ultimate cure of the hernia depends upon many considerations, such as age of the patient, size of the openings, nature of the operative procedure, and after-treatment of the patient. The best statistics give for under twenty-five years of age, fully 62 per cent. of permanent cures; in those above this age, 42 per cent. The method of operating adopted by Dr. Fowler is as follows: The sac is first opened and carefully explored, to ascertain that no adherent bowel or omentum occupies its canal. The neck of the sac is then tied, either by simply encircling it, or by a line of through-and-through stitches, in cobble-stitch fashion, chromicised gut being the material made use of in either case. The sac is then cut away beyond the ligature, and the stump allowed to fall within the internal ring. The next point is such a disposition of the spermatic cord as will permit of complete obliteration of the inguinal canal and closure of the ring. For this purpose he employs Bassini's method, modified by Postempski. This consists essentially in displacing the cord in a direction toward the median line, lifting it for this purpose from the canal after completely freeing it, and attaching it by loose loops of buried catgut suture to the abdominal wall beneath the skin. In order to still further counteract any tendency of new protrusion occurring and following the cord, he forces the cord at a point where it emerges from the ring well up into a slit made

[1] *Annals of Surgery*, vol. xvii., part i.

for the purpose in the upper margin of the latter, fixing it by a loose loop of catgut. The canal is then closed by "a crossed suture." The material employed is *crin de Florence*, or silk-worm gut, which is threaded at both ends upon large and full-curved Hagedorn needles. These are passed from behind forward, one through each edge of the divided lowermost layer. The latter consists essentially of conjoined tendon upon the inner margin, and Poupart's ligament upon the outer. The needles, after emerging each from its respective side, are reversed as regards position, that which passed through Poupart's ligament being now carried to the inner side and passed through the skin, again from behind forwards, while that which included the inner margin of the lowermost layer is passed through the skin at the outer margin in the same manner. By tying the sutures over the skin the incision is completely closed. The sutures are placed about three-eighths of an inch apart, and a sufficient number are employed to completely close the wound, no drainage being employed. The sutures are left in place for three weeks, and then removed by cutting upon one side of the knot and drawing them out. The patient thus operated upon is never permitted to sit up in bed for the first six weeks following the operation. No truss is subsequently worn.

Dr. Henry O. Marcy, of Boston, has collected statistics[1] showing the result of operations for the radical cure of hernia. In all he has collated 3,000 cases, and the proportion of deaths was less than 1 per cent. Among them are—

| | | | | | | |
|---|---|---|---|---|---|---|
| Bassini | ... | 262 operations | ... | 1 death. | | |
| Championnière | | 254 ,, | ... | 2 deaths. | | |
| Schede | .. | 165 ,, | ... | 2 ,, | | |
| Banks | ... | 106 ,, | ... | 0 ,, | | |
| Park | ... | 115 ,, | ... | 0 ,, | 85 cured. | |
| Marcy | ... | 115 ,, | ... | 0 ,, | 4 relapses. | |

He considers the essentials of the operation to be—

1. Strict aseptic conditions.

2. A free dissection, in order to lay bare the internal ring, permit of the enucleation of the peritoneal sac, and the separation and elevation of the cord out of the wound.

3. The separation of the sac to its very base before removal. The sac should always be opened, and then tension should be made upon it, and sutures applied in the line of the long diameter of the internal ring; it should then be resected near its base.

4. Having freed the cord to its point of entrance within the abdominal cavity, and lifted it to one side, a row of continuous tendon sutures is inserted from below upwards until the internal ring is closed upon the cord at its exit from the abdominal cavity. The external structures are then united in

[1] *New York Med. Journ.*, Feb. 4, 1893.

the same manner with a deep double layer of tendon sutures, joining the divided muscular wall of the abdomen, and bringing into close apposition Poupart's ligament and the conjoined tendon until the external ring is reconstructed. The structures external to the muscles are approximated by one or more layers of single continuous sutures taken by means of a Hagedorn needle introduced from side to side, and in a similar manner the skin is closed with a continuous buried tendon suture. The entire operation is conducted under the irrigation of a weak sublimate solution, and the parts are afterwards dusted with iodoform before sealing with collodion. No drainage tubes are used.

The following methods of performing the radical cure of femoral hernia are quoted by Salzer[1]: Billroth sews the middle third of Poupart's ligament to the deep fascia, or unites it to the middle septum of the femoral sheath, using a triple silk suture. Czerny uses catgut: Schede, catgut, silk, or silver wire; the latter he has used since 1887, and finds it more certain, and also that it may remain *in situ* without harmful results. He had one relapse in four cases which he had under observation, the relapse occurring after five years. Lauenstein united the falciform process and Gimbernat's ligament after folding the sac in the manner employed by Macewen in inguinal hernia. Bergen fixes the ligated stump of the hernial sac above Poupart's ligament, and sutures the ligament to the aponeurosis of the pectineal muscle, producing a direct union. He advises that the thigh be flexed upon the pelvis during healing.

The author believes that in all these methods, where tension is employed by means of sutures, the resulting scar-tissue is of doubtful utility, as has been shown in the relapse in Schede's case after five years, and advocates the following method, which he has employed successfully: A curved incision is made in the pectineal fascia beneath the femoral vessels, extending from near the crista pectinea to Gimbernat's ligament; a flap is dissected up, its base proximal and joining Gimbernat's ligament; the free or distal border he unites by silk sutures, without tension, to the middle third of Poupart's ligament. Thus is formed a strong fibrous septum, which he deems a practical and certain method for the radical cure of these herniæ. His researches on the cadaver have shown that the pectineal fascia varies in thickness, and his observations lead him to believe that it is thickened in persons who have worn a truss, and in general is thicker in adults.

W. H. Harsant.

[1] *Centralbl. für Chir.*, 1892, No. 33, abstracted in *Amer. Journ. Med. Sci.*, Jan., 1893.

